# Evolution of Physicochemical Properties, Phenolic Acid Accumulation, and Dough-Making Quality of Whole Wheat Flour During Germination Under UV-B Radiation

**DOI:** 10.3389/fnut.2022.877324

**Published:** 2022-04-28

**Authors:** Chao Tian, Yue Wang, Tianbao Yang, Qingjie Sun, Meng Ma, Man Li

**Affiliations:** ^1^School of Food Science and Engineering, Qingdao Agricultural University, Qingdao, China; ^2^Food Quality Laboratory, Beltsville Agricultural Research Center, United States Department of Agriculture-Agricultural Research Services, Beltsville, MD, United States

**Keywords:** UV-B radiation, germinated wheat, phenolic acids, ascorbic acid, suppress deterioration

## Abstract

The effects of ultraviolet-B (UV-B) radiation on the physiological properties, phenolic acid accumulation, and dough-making quality of wheat during germination were investigated. UV-B radiation inhibited the wheat sprout length and reduced the dry matter loss. As phenolic acids were principally present in the kernels' bran, UV-B radiation could promote their accumulation in the interior of germinated wheat (GW). The total phenolic compounds, ascorbic acid, and antioxidant activity were also enhanced significantly during germination with UV-B. UV-B improved the development time, stability time, rheological properties, and viscosity of GW, and inhibited the α-amylase activity, the destruction of the amorphous region of starch particles, and the proteins degradation process during germination, and thus the deterioration of dough-making quality caused by germination was inhibited. Therefore, UV-B radiation could be a potential approach to enhance the nutritional and dough-making quality of germinated whole wheat flour.

## Introduction

Whole grains contain unique bioactive ingredients that promote health, presenting a more complex and beneficial nutritional profile than refined grains. A moderate daily intake of whole-grain foods has been linked to lower blood pressure, which in turn reduces the risk of cardiovascular disease (1). Among whole grains, whole wheat occupies an important position and contributes a lot to human food.

The processing of whole wheat flour can affect the composition and nutritional value of whole wheat products (2). Compared with refined wheat flour, whole wheat flour contains more vitamins, minerals, dietary fibers, and other nutrients and helps control the rise of blood sugar, whose glycemic index is lower than that of refined wheat flour (3, 4). The consumption of whole wheat has been on the rise in recent years as people seek to get more nutrients from food as they pursue a sense of satiety (5).

Germination is the process of enhancing the bio-accessible minerals and nutrient content absorbed by the human body in plant foods (6). It can increase the content of total protein and essential amino acids of soybean (7). Germination increased the antioxidant activity of legumes such as chickpeas, lentils, and yellow peas (8). However, for wheat, excessive germination will lead to the degradation of gluten, which has a negative impact on the processing quality (9).

Ultraviolet-B (UV-B) radiation on wheat kernels during germination may weaken the adverse effects of germination on the processing quality of whole wheat flour. The effect of UV-B radiation, which is considered as stress on plant seed germination and growth, is a hot area of research at present, and the impact mechanisms of plants in response to UV-B stress are also different. UV-B stress will affect plant growth, from the morphology of plant parts to physiological and biochemical nutrients, and other aspects. UV-B radiation makes a difference to plant morphology and structure, physiological metabolism, and growth state (10). So far, UV-B radiation, as a benign inducer in plants, has been studied to induce the accumulation of some active substances such as isoflavones (11).

Although UV-B radiation has been proven to exert influence on plant growth, the mechanism of the effects of germination and UV-B radiation on the physiological activity, phenolic acid content, and dough-making quality of whole wheat flour is not clear. To obtain higher nutrition and better processing quality of whole wheat flour, we studied the evolution of the nutritional composition and quality characteristics of germinated whole wheat flour during UV-B radiation.

## Materials and Methods

### Materials

Wheat (*Triticum aestivum* L. cv. Zhongmai 998) was cultivated by the Hebei Woyu Agriculture Technology Co., Ltd. (Shijiazhuang, Hebei, China).

### Wheat Germination Process

**Figure 1:**
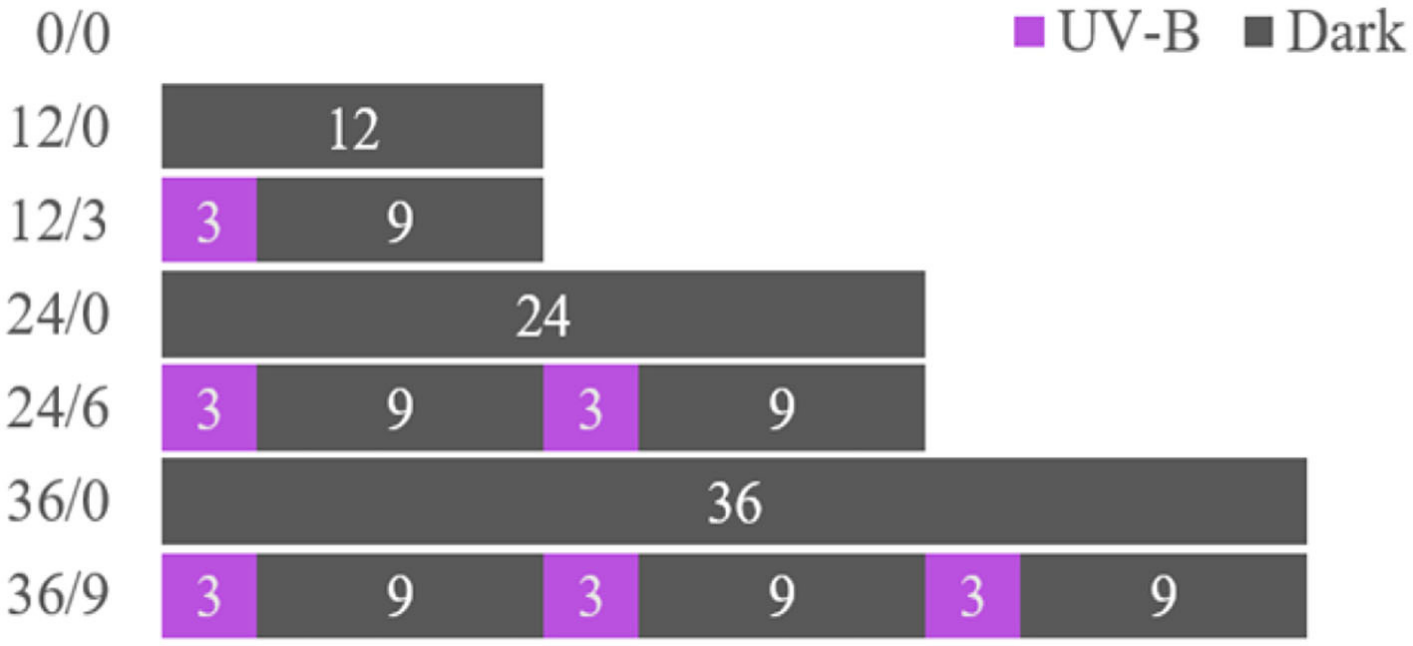
Experimental scheme for the preparation of wheat kernels treated with UV-B. Germinated wheat was treated with UV-B radiation (0, 3, 6, and 9 h) over different time intervals (0, 12, 24, and 36 h).

The wheat kernels were sieved, cleaned, and soaked in distilled water (25°C) for 6 h, then placed on a tray lined with wet gauze in a constant temperature and humidity incubator (PCTHI-250T, STIKCO., LID.) for germination [30°C, 80% relative humidity (RH)]. Pure water was sprayed every 6 h to keep the gauze moist and the wheat kernels germinated for 0, 12, 24, and 36 h, respectively. Some soaked kernels were germinated under dark, and others were radiated by UV-B (10 μW/cm^2^) for 3 h every 12 h period (11, 12). The details of the experiment design are shown in Figure 1. Germinated wheat (GW) under different radiation times were collected on the fixed day. Ungerminated wheat (UW) after soaking and GW were dried at 45°C to a moisture content (mc) of 12–14% in the electric heating constant temperature blast drying oven. (During the test, the experimenter should use good personal protection and avoid direct contact with UV-B).

### Measurement of Sprout Length and Dry Matter Loss

The sprout lengths of wheat kernels (30 granules) at different germination and radiation time were measured using a vernier caliper with three repeats. Dry matter losses were calculated according to the difference in dry matter weight between dried UW and GW, with three repeats, and 100 granules were measured for each repeat.

### Wheat Milling

Wheat kernels were milled in an MLU202 test mill (MLU-202, Buhler Machinery Manufacturing Co., Ltd.) to obtain 1B, 2B, 3B, 1M, 2M, and 3M coarse bran and fine bran flours. 1B, 2B, and 3B streams belonging to the break system were the flour obtained by milling the wheat 1, 2, and 3 times with the break roll of the above test mill, respectively. 1M, 2M, and 3M streams belonging to the milling system were the flours milled 1, 2, and 3 times with the milling roll from the fractions that could not be sufficiently milled with the break roll, respectively. 1B, 2B, 3B, 1M, 2M, and 3M coarse bran and fine bran flours of all samples were mixed as whole wheat flour. The flours were placed at −18°C for subsequent analysis.

### Distribution, Compositions, and Contents of Free and Bound Phenolic Acids

Different layers of flours were divided into three groups (coarse bran and fine bran were mixed into the bran flour; 1B, 2B, and 3B were mixed to form the break flour; and 1M, 2M, and 3M were mixed to form the milling flour). Different groups of wheat flour samples (2 g) (bran flour, break flour, and milling flour) were extracted with 80% methanol (10 ml) for 3 times (200 rpm, 1 h), respectively, under the dark (sealed with nitrogen at 25°C). Then the extracted solutions were centrifuged (1,000 rpm, 15 min, 4°C) and the supernatants extracted three times were mixed and rotatably evaporated to dry at 40°C. Next, they were dissolved in 50% methanol (10 ml) and used as free phenolic extracts at constant volume. After being filled with nitrogen, the samples were stored at −18°C for subsequent research.

The precipitates obtained by centrifugation three times were mixed and hydrolyzed by 20 ml sodium hydroxide (2 mol/L) under dark (25°C, 4 h). The pH of the hydrolyzed solutions was adjusted with a hydrochloric acid solution (6 mol/L) at a range of 1.5–2.0. Then 25 ml ethyl acetate was mixed with the solution for 15 min and centrifuged (1,000 rpm, 15 min, 4°C, repeated extraction three times). The upper layers of ethyl acetate were rotatably evaporated to dry at 40°C and then dissolved with 50% methanol (10 ml). After being filled with nitrogen, all samples were stored at −18°C. Phenolic acid contents were determined by the high-performance liquid chromatography (HPLC) (Shimadzu LC-20A, Kyoto, Japan) and loaded on a Gemini C18 column (Phenomenex, America) at 280 nm (13).

### Determination of Ascorbic Acid

The contents of AsA in the ungerminated and germinated whole wheat flour were measured using the method of dinitrophenylhydrazine (14). The extract was a solution of 3% metaphosphoric acid and 8% acetic acid. The UW and GW flour samples (0.5 mg) were dissolved in an extract solution (8 ml) and centrifuged. The bromine water (0.115 ml) was added to the supernatant (2 ml), then mixed with 0.065 ml of 10% thiourea, 0.5 ml of 2,4-dinitrophenylhydrazine (DNPH), and reacted at 37°C for 3 h. After the above solutions were cooled in an ice bath, 80% sulfuric acid solution (2.5 ml) was added to them. The absorbance of solutions was measured at 521 nm.

### Determination of Total Phenolic Compounds

The contents of TPC in UW and GW flour were analyzed using Folin–Ciocalteu's phenol reagent (15). The absorbances were measured at 739 nm. The freshly prepared gallic acid solution (1 mg/ml) was used as a standard, and the results were expressed as mg of gallic acid equivalents (GAE) per 100 g dry matter.

### DPPH (1,1- Diphenyl-2-Picrylhydrazyl) Radical Scavenging Activity

The whole wheat flour was evaluated spectrophotometrically by their shoot. According to the method described by Pajak et al. (16), UW and GW flours (1 g) were dissolved in 10 ml pure methanol, extracted for 1 h (25°C), and then centrifuged (3,500 r/min, 10 min, 25°C). Supernatants of the samples (0.1 ml) were mixed with 3.9 ml DPPH radical solution (0.1 mM) made in methanol freshly, respectively. Next, we measured the absorbance of the mixed solution as A_0min_ at 515 nm in a UV/Vis spectrophotometer. After 30 min of incubation, the absorbance of the sample was measured as A_30min_.


$$\begin{array}{l} \text{DPPH radical scavenging activity}(\%)\;\\ \text{ =}\frac{{\text{A}}_{\text{0min}}{\text{− A}}_{\text{30min}}}{{\text{A}}_{\text{30min}}}\times 100\% \end{array}$$

### Quality Evaluation of Whole Wheat Flour

#### Measurement of the Mixing Properties

The whole wheat flour was added into the farinograph equipped with a mixing bowl (Brabender, Duisburg, Germany) in the corresponding quantity according to the water content (ICC 115/1). The thermostat with a circulating water pump kept the temperature of the bowl steady at 30°C. Development time, stability time, and degree of softening of wheat dough were determined.

#### Dynamic Rheological Properties

The dynamic rheological properties of whole wheat flour were measured using a dynamic rheometer (MCR102, Anton Paar, Vienna, Austria). UW was used as control. The whole wheat flour with different radiation and germination time was mixed with water (64 g/100 g flour) to prepare dough. Dough samples (3 g) were placed between the plates after 10 min of wakening to eliminate the stress generated. Before the test was started, the edges of the excessive dough samples were scraped off with a plastic sheet and were applied a thin layer of liquid paraffin wax. The frequency sweep was carried out at a frequency of 0.1–100 Hz at 25°C, using parallel plate geometry (40 mm diameter and a gap of 2 mm).

#### Rapid Visco Analyzer Analyses

The gelatinization properties of whole wheat flour were measured by a Rapid Visco Analyzer (S/N 2194407-TMB, perten, Australia). The suspensions were prepared from pure deionized water (25 g) and whole wheat flour (3.5 g), which were manually homogenized using a plastic paddle. The tests were performed in a programmed heating and cooling cycle.

#### Thermal Properties of Whole Wheat Flour

Thermal properties of whole wheat flour were analyzed to evaluate gelatinization temperature and enthalpy. Every flour sample (~4 mg) and deionized water with a water-sample ratio of 2:1 were precisely weighed and kept in a sealed DSC pan. All pans were equilibrated at 25°C for 12 h before being analyzed and then heated from 25 to 105°C.

#### X-Ray Diffraction

The X-ray diffractometer equipped with a divergence slit (D8 Advance, Bruker, Germany) was operated at 40 kV and 40 mA. All whole wheat samples were scanned from 4° to 40° (2θ) at a speed of 2°/min and a step size of 0.02°. The diffractograms were analyzed and the relative crystallinities were calculated using the software of MDI Jade 6.5. The relative crystallinity (RC) (%) was calculated using the percentage ratio of the crystalline and amorphous regions. The RC of the samples was calculated using the following equation:


$$\begin{array}{l} Relative\;crystallinity\;\left(\%\right)=100Ac/\left(Ac+Aa\right) \end{array}$$

where Ac is the area of the crystalline peak and Aa is the area of the amorphous peak.

#### Size Exclusion High-Performance Liquid Chromatography

The molecular weight distribution of wheat proteins was conducted by the SE-HPLC system (LC-20AT, Shimadzu, Japan) equipped with a UV detector. All whole wheat flour samples (15 mg) were extracted with 1 ml phosphate buffer saline (PBS, 0.05 M, pH 7.0) consisting of 1.0% SDS (w/v). All solutions were stirred with a constant temperature mixer (25°C, 30 min, 1,500 rpm) (MSC-100, ALLSHENG, Hangzhou). After centrifugation (10 min, 11,000 *g*), the supernatants (20 μl, filtered through a 0.45 μm filter) of each sample were injected into a TSK G4000-SWXL column (Tosoh Biosep, Japan). The PBS solution was achieved as elution of the samples with a flow rate of 0.7 ml/min, at 30°C, 214 nm signal intensity (17).

#### Protein Solubility

Whole wheat flour samples (1 g) were added to distilled water (50 ml) and the suspensions were adjusted to pH 6.0 by 0.1 mol/L HCl and NaOH. Next, the suspensions were shaken adequately and centrifuged (8,000 *g*, 15 min) to obtain the supernatants (18). Protein solubility was determined by assaying the protein contents in the supernatants.

### Statistical Analysis

All results were expressed as the mean ± standard deviation. Statistical analysis was carried out using SPSS 20.0 (SPSS Inc., Chicago, USA). All data were means of at least three measurements. Significance was determined by one-way analysis of variance (ANOVA) and Duncan's test, and the *P*-value of < 0.05 was statistically significant.

## Results and Discussion

### Effect on Sprout Length and Dry Matter Loss

**Figure 2:**
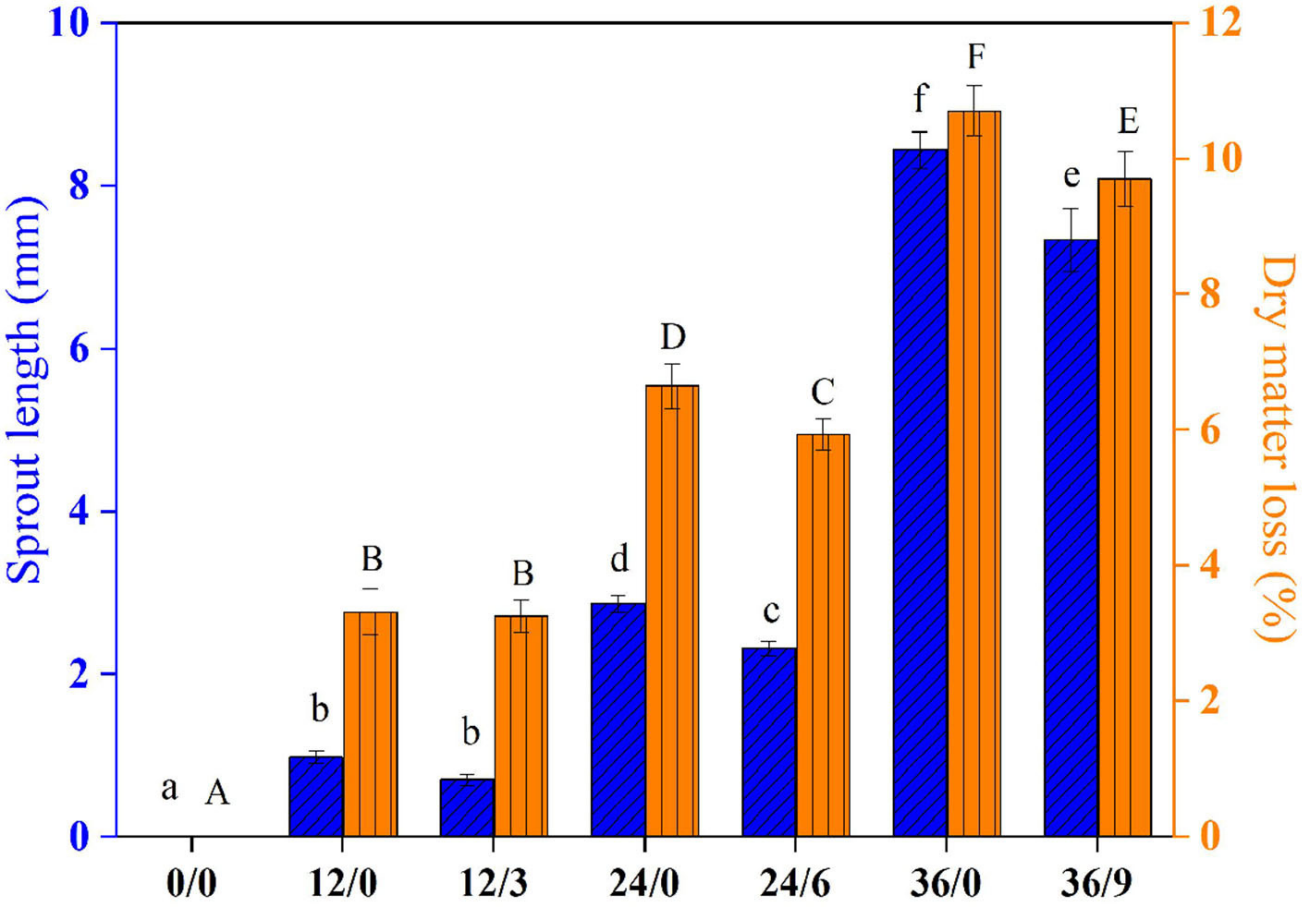
The sprout length and dry matter loss of wheat during germination under UV-B radiation. Different letters indicate the results of Duncan's multiple range test at 5% level of significant difference among different germination and UV-B radiation time, respectively.

As shown in Figure 2, UV-B radiation resulted in shorter sprout lengths. UV-B radiation for wheat kernels 6 h (germinated 24 h) and 9 h (germinated 36 h) had a significant inhibition effect on sprout length. With increasing germination time, the dry matter loss of wheat kernels increased, which might be due to the consumption of some nutrients such as storage protein and sugars in seeds caused by enhanced respiratory metabolism during germination (19). However, the dry matter loss of wheat kernels after 24 and 36 h germination was significantly inhibited by UV-B radiation, from 6.64% and 10.70% to 5.93% and 9.69%, respectively. It might be because UV-B suppressed the growth process and respiratory metabolism, and then reduced the consumption of nutrients such as storage starch and protein during wheat growth.

### Effects on Phenolic Acid Contents

**Figure 3:**
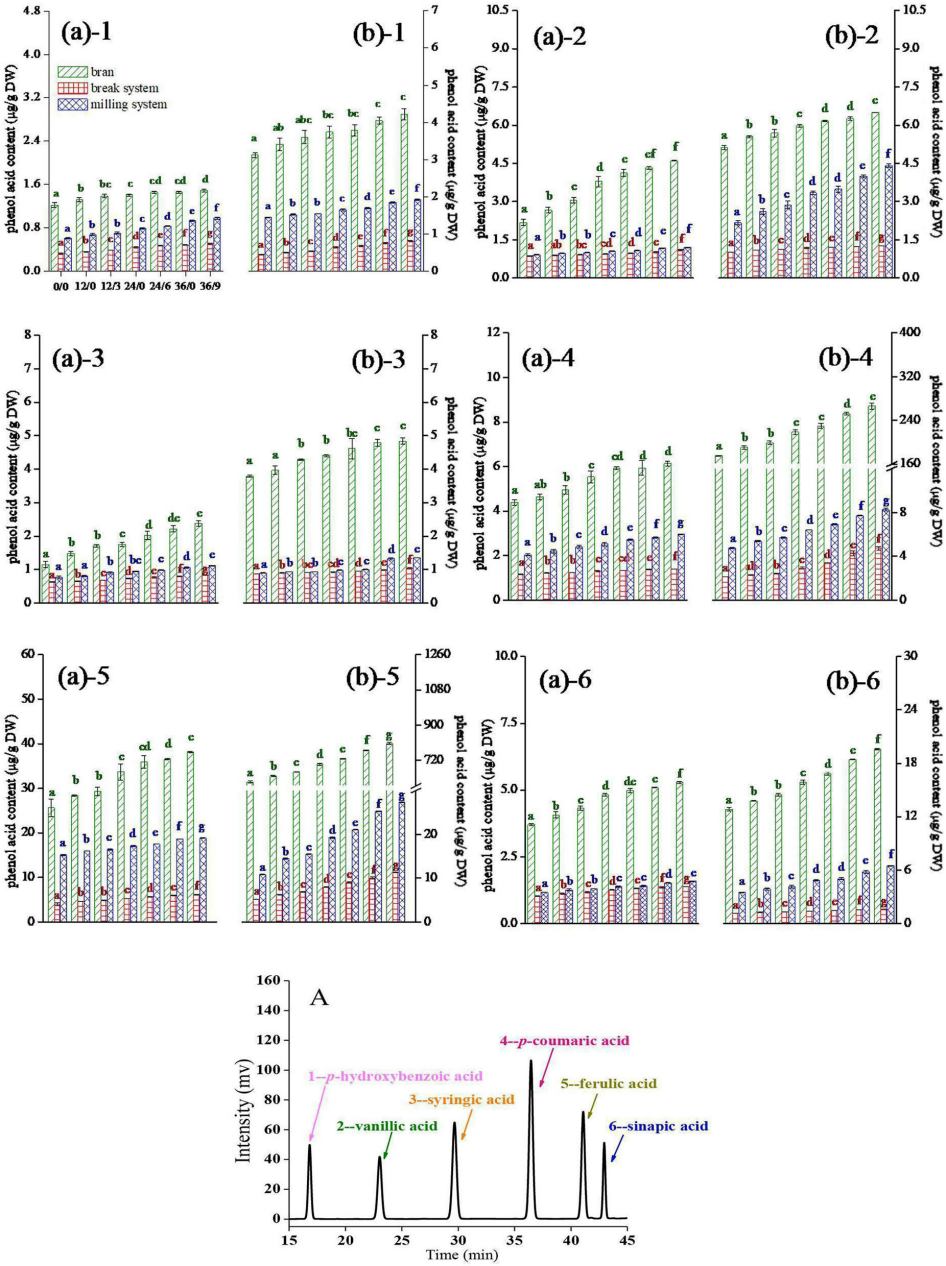
1–*p*-Hydroxybenzoic acid, 2–vanillic acid, 3–syringic acid, 4–*p*-coumaric acid, 5–ferulic acid, and 6–sinapic acid (in the order of retention time) contents of wheat during germination under UV-B radiation. **(A)** free phenol acid; **(B)** bound phenol acid. Bran indicates the bran flours, which is a mixture of coarse bran flours and fine bran flours. Break indicates the break system flours, which is a mixture of 1B, 2B, and 3B flours. Milling indicates the milling system flours, which is a mixture of 1M, 2M, and 3M flours. A: HPLC chromatogram of standards of six phenolic acids. Different letters indicate the results of Duncan's multiple range test at 5% level of significant difference among different germination and UV-B radiation time, respectively.

The contents of free and bound phenolic acids in different layers of wheat flour are shown in Figure 3. Six main phenolic acid monomers in wheat were identified: ρ-hydroxybenzoic acid (1), vanillic acid (2), syringic acid (3), ρ-coumaric acid (4), ferulic acid (5), and sinapic acid (6), which was consistent with Chen et al. (13).

As shown in Figure 3, phenolic acids mainly existed in the form of bound states in wheat, which was consistent with Chen et al. (20). The contents of free and bound phenolic acids in wheat flour were increased during germination and further enhanced after UV-B radiation, and the increase rate of the bound states was fastest after UV-B radiation, which was due to UV-B radiation could activate the defense system of GW. Phenolic acid produced through the phenylpropane metabolic pathway can directly scavenge active oxygen free radicals to resist the damage of UV-B radiation to cells (11). Phenolic acid mainly existed in the bran flour, followed by the milling flour, and the break flour was the least. The accumulation of phenolic acids in the bran layer was facilitated by UV-B radiation, which was due to UV-B radiation first acted on the bran layer and promoted the deposition of phenolic acid in the cell wall of the wheat epidermis. The content of free and bound phenolic acids continued to increase in break and milling systems, which might be due to UV-B radiation penetrating the bran layers to reach the inside of the wheat kernels and activated genes and enzymes related to phenylpropanoid metabolism in wheat.

After a longer germination time (36 h), the increase rate of *p*-hydroxybenzoic acid, vanillic acid, and syringic acid (Figure 3(a)1-(a)3, 2(b)1-(b)3) in the bran layer was not significant, which might be due to that some cells in wheat bran layer were damaged by the high dose of UV-B during continuous radiation, and the accumulation process of the three phenolic acids in wheat bran layer was adversely affected. As shown in Figure 3(a)4-(a)6, 3(b)4-(b)6, the contents of *p*-coumaric acid, ferulic acid, and sinapic acid in wheat bran increased continuously with germinating time and UV-B radiation time. Hilal et al. (21) reported lignin deposition in epidermal tissues was a resistance mechanism against UV-B radiation. In addition, the main building blocks of lignin originated from ρ-coumaric acid, ferulic acid, and sinapic acid through a series of biochemical reactions (22). Therefore, we concluded that the continuous increase in ρ-coumaric acid, ferulic acid, and sinapic acid contents in wheat bran powder was related to the change of lignin under UV-B radiation.

### Effect on AsA

**Figure 4:**
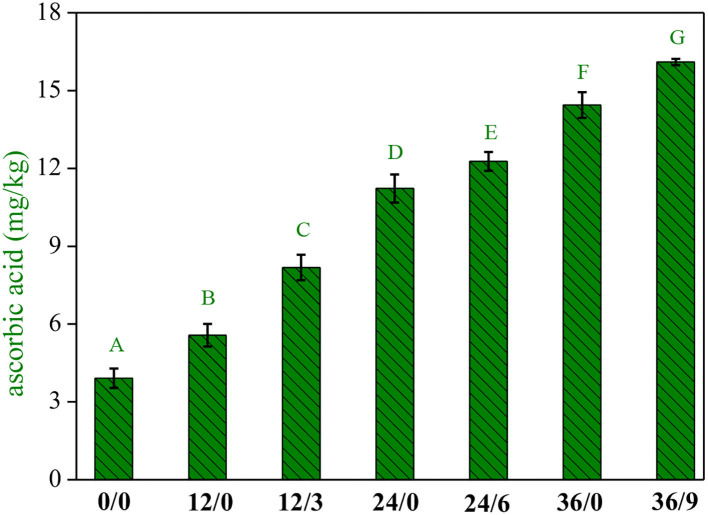
The ascorbic acid contents of whole wheat flour during germination under UV-B radiation. Different letters indicate the results of Duncan's multiple range test at 5% level of significant difference among different germination and UV-B radiation time, respectively.

UV-B radiation and germination time interacted significantly on AsA accumulation (Figure 4). The content of AsA in wheat kernels was low but increased significantly with the extension of germination time, which increased by 1.4, 2.8, and 3.6 times when germinated for 12, 24, and 36 h, respectively. It was further increased by 2.1, 3.1, and 4.1 times with UV-B radiation. Kaushik et al. (23) reported that the AsA contents of soybean enhanced after germination. Xu et al. (24) showed that AsA levels in soybean sprouts increased significantly with ultraviolet radiation, which might be related to the reactivation of biosynthesis during wheat germination, in which the activation of galacturonic dehydrogenase, a key enzyme catalyzing the formation of AsA, increased AsA content (14).

### Effect on TPC and DPPH (1,1-Diphenyl-2-Picrylhydrazyl) Radical Scavenging Assay

**Figure 5:**
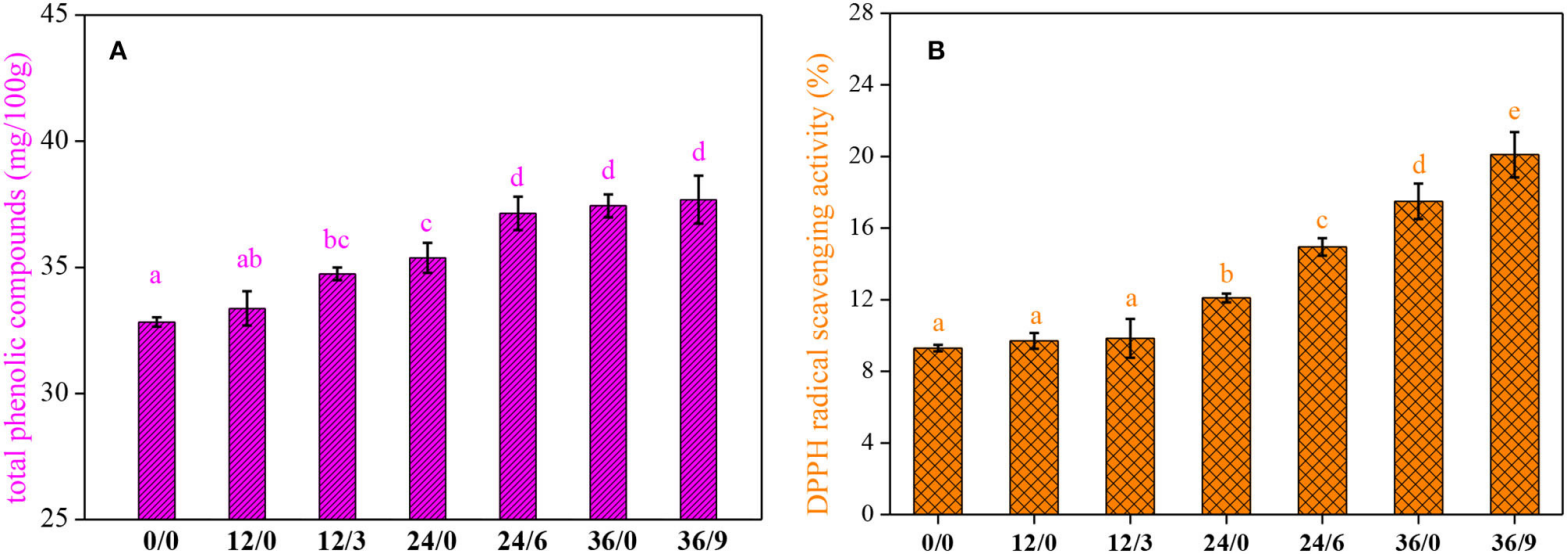
The total phenolic compounds **(A)** and DPPH free radical scavenging capacity **(B)** of whole wheat flour during germination under UV-B radiation. Different letters indicate the results of Duncan's multiple range test at 5% level of significant difference among different germination and UV-B radiation time, respectively.

Germination generated an increase in TPC (*P* < 0.05), and the effect was time-dependent. As shown in Figure 5A, germination could increase the TPC of whole wheat flour, and UV-B radiation facilitated the accumulation of phenolic substances in a short period. The increase in TPC of germinated whole wheat flour may be put down to the activation of phenylalanine ammonia-lyase (the key enzyme in phenolic biosynthesis), which contributed to the formation of phenolic compounds during germination (25). In addition, UV-B radiation could regulate phenylalanine ammonia-lyase activity (26).

The DPPH radical-scavenging activity increased significantly during germination (Figure 5B), almost 190% higher when germination for 36 h, and further increased to 216% with UV-B radiation. It was consistent with the changing trend of phenolic acid, TPC, and ASA. These components were enhanced and displayed the enhanced antioxidant activity through scavenging free radicals in the process of germination and UV-B radiation.

### Mixing Properties

**Figure 6:**
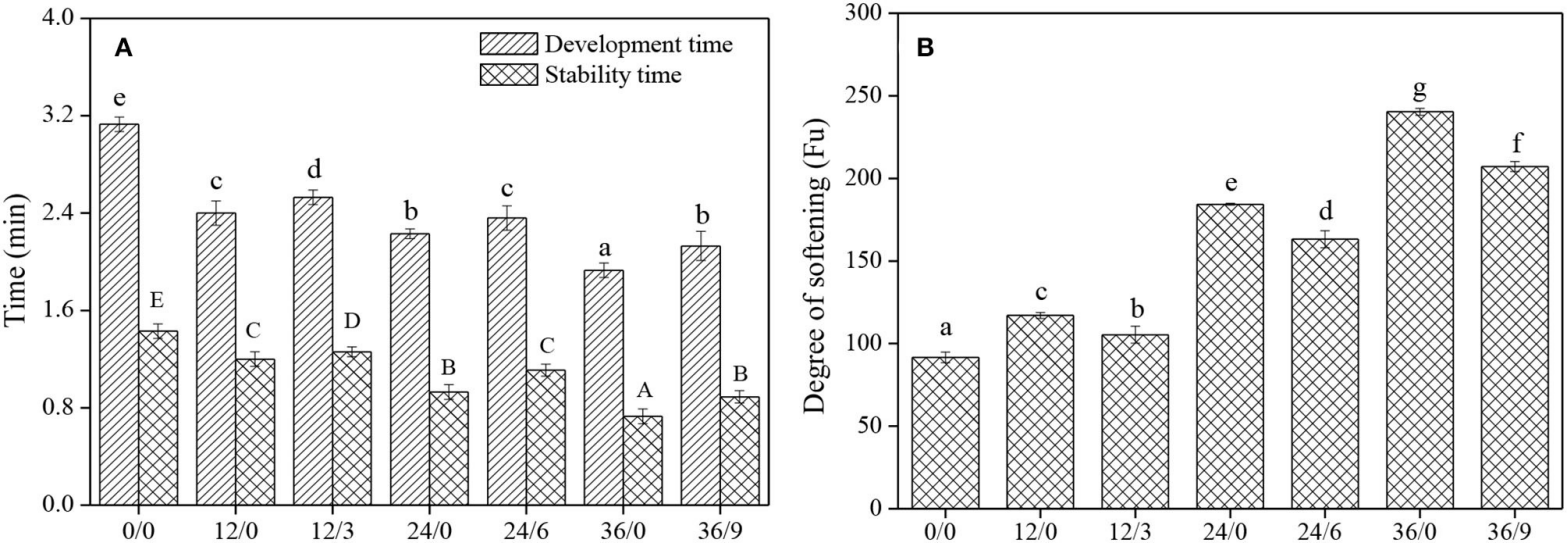
The development time, stability time and degree of softening of whole wheat flours during germination under UV-B radiation. Different letters indicate the results of Duncan's multiple range test at 5% level of significant difference among different germination and UV-B radiation time, respectively.

The mixing properties of wheat dough reflected the resistance of dough to mechanical agitation. Wang et al. (27) reported that the development time and stability time of whole wheat flour were lower than that of traditional refined flour, possibly because the bran portion of whole wheat flour attenuated the gluten and thus impaired the strength of the gluten network. The development time and stability time of whole wheat flour decreased after germination (Figure 6A), possibly owing to the enhancement of amylase activity, then the destruction of starch granules, and the weakening of the binding between starch and protein matrix during germination. The development time and stability time of whole wheat flour were slightly prolonged under UV-B radiation (Figure 6A) (the development time and stability time of 36/9 were 10.36% and 21.92% higher than that of 36/0), and the weakening degree of whole wheat flour was significantly inhibited (Figure 6B) (the weakening degree of 36/9 was 13.77% lower than that of 36/0), which might be because UV-B radiation could inhibit the germination of wheat kernels, then the amylase activity was inhibited and dough weakening was prevented.

### Rheological Analysis

**Figure 7:**
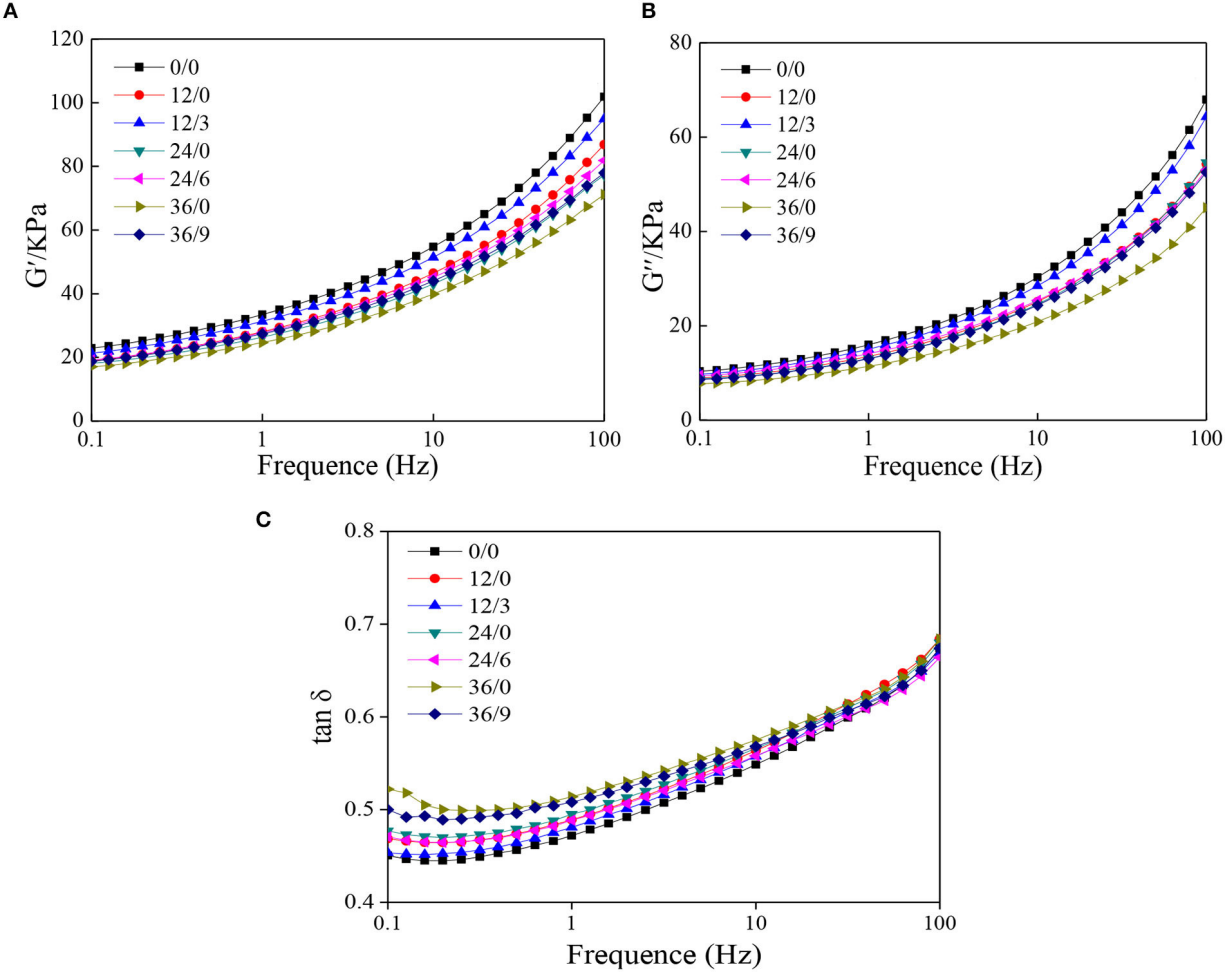
The dynamic rheological properties of whole wheat dough during germination under UV-B radiation. **(A)** storage modulus (G′); **(B)** loss modulus (G"); and **(C)** loss tangent (tanδ).

In defining the rheological behavior of the dough, starch and gluten are important molecules from flour. Among the main compositions of the flour, starch is mainly known to behave as a nonlinear viscoelastic response, while it is reported that gluten is responsible for dough elasticity, being highly resistant to stress sweep (28). The storage modulus (G′) of the wheat dough gives the concept of the deformation energy stored in the dough after oscillation and provides its elastic behavior. The large storage modulus of dough indicates good elasticity. In contrast, loss modulus (G") stands for the energy lost by the dough during oscillation. The large loss modulus of dough indicates good viscosity (29). As shown in Figures 7A,B, G′ of all dough samples was higher compared to G", which indicated the solid-like property of the samples. However, G′ and G" of dough decreased after germination, which indicated the viscoelasticity of dough decreased. It was consistent with Wang et al. (30). G′ and G" were higher with UV-B radiation, which indicated the decreases in viscoelasticity of dough were alleviated under UV-B radiation. It may be because UV-B radiation inhibited the decomposition of macromolecular proteins during germination.

Loss tangent (tanδ) was one of the important indexes to measure the quality of wheat gluten. The higher the value of tanδ, the higher the degree of deterioration of wheat gluten. Therefore, Figure 7C indicated that the degree of gluten deterioration was higher after germination but inhibited by UV-B radiation.

### Viscosity Analysis

**Figure 8:**
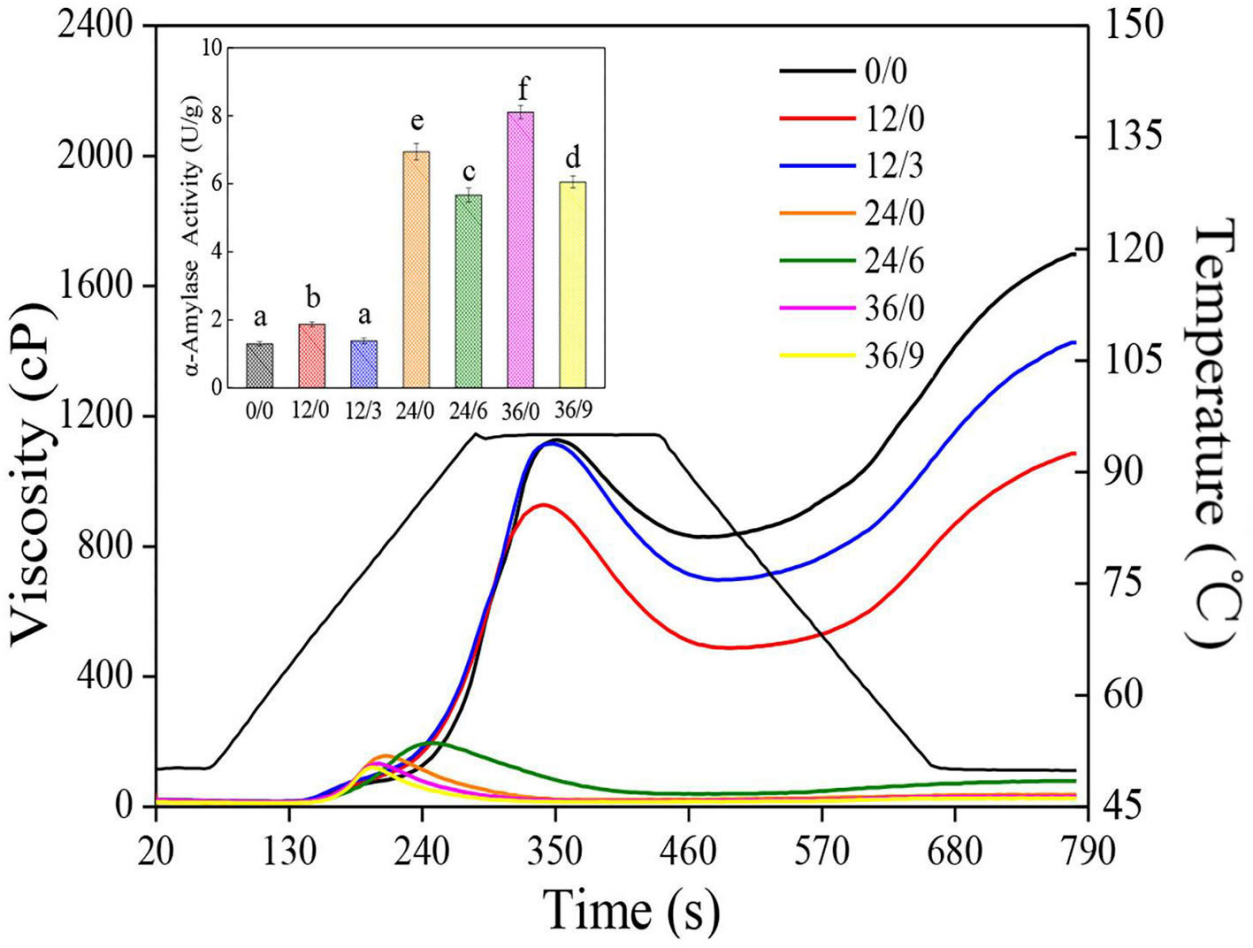
The pasting properties of whole wheat flours during germination under UV-B radiation. The inset shows α-amylase activity of wheat. Different letters indicate the results of Duncan's multiple range test at 5% level of significant difference among different germination and UV-B radiation time, respectively.

The α-amylase is commonly deemed as one of the principal factors for wheat flour production, which can hydrolyze large starch molecules, resulting in a dramatic reduction in viscosity (31). As shown in the inset of Figure 8, the α-amylase activity of wheat increased sharply after germination, which increased to 8.11 U/g after germination for 36 h while significantly inhibited with UV-B radiation, which was 25.4% lower in 36/9 than in 36/0. Increases in α-amylase activities in GW were directly concerned with the hydrolysis of starch in whole wheat flour, which led to the deteriorative processing qualities. Therefore, the reduction of α-amylase activities under UV-B radiation was probably conducive to the better whole wheat flour quality.

Furthermore, the influence of germination and UV-B radiation on gelatinization characteristics (RVA parameters) is shown in Figure 8. Pasting properties are generally concerned with the swelling and rupture of starch granules. The peak viscosity, breakdown, trough viscosity, final viscosity, setback, and peak time decreased significantly during germination (Figure 8), which might be due to the enhancement of α-amylase activity, resulting in the hydrolysis of large starch particles, then reducing the hardness of starch particles, leading to a faster expansion rate of starch (27). The reduction in the peak viscosity of whole wheat flour might be due to the degraded starch and the reduced swelling index of starch caused by germination. The lower setback and final viscosity were primarily because of the reordering of amylose and amylopectin, which caused a reduction by the degradation of amylopectin branched chains (32). Nonetheless, the decline of viscosity was inhibited under UV-B radiation, which was possible because UV-B radiation inhibited the enhancement of α-amylase activity and then slowed down the tendency of starch hydrolysis to a certain extent, thereby slowing down the deterioration of the gelatinization properties.

### XRD and Thermal Characteristics Analysis

As shown in Table 1, there is no significant effect of germination and UV-B radiation on the RC of whole wheat flour. Combined with the results of the viscosity characteristics (RVA) of UW and GW flour, we speculated that the decrease in the viscosities of whole wheat flour was not related to the crystallization zone of wheat starch but to the amorphous zone of starch grains hydrolyzed by α*-*amylase.

**Table 1:** The relative crystallinity and thermal parameters (To, Tp, Tc, and ΔH) of whole wheat flour during germination under UV-B radiation.

**Samples**	**RC (%)**	**To (**°**C)**	**Tp (**°**C)**	**Tc (**°**C)**	**ΔH (J/g)**
0/0	19.70 ± 0.18^a^	64.53 ± 0.01^e^	71.54 ± 0.07^c^	68.02 ± 0.01^d^	4.93 ± 0.11^c^
12/0	18.87 ± 1.25^a^	63.50 ± 0.36^de^	70.78 ± 0.42^bc^	67.00 ± 0.45^cd^	4.45 ± 0.28^bc^
12/3	21.01 ± 2.17^a^	63.57 ± 0.52^de^	70.87 ± 0.40^bc^	67.06 ± 0.34^cd^	4.67 ± 0.20^bc^
24/0	19.64 ± 0.35^a^	62.13 ± 0.25^bc^	70.19 ± 0.36^ab^	66.06 ± 0.13^abc^	4.31 ± 0.06^bc^
24/6	20.34 ± 0.66^a^	62.70 ± 0.99^cd^	70.21 ± 1.12^ab^	66.39 ± 1.09^bc^	4.34 ± 0.55^bc^
36/0	18.32 ± 0.35^a^	60.87 ± 0.01^a^	69.02 ± 0.13^a^	65.01 ± 0.01^a^	3.65 ± 0.09^a^
36/9	20.34 ± 2.28^a^	61.09 ± 0.14^ab^	69.50 ± 0.11^a^	65.41 ± 0.07^ab^	4.16 ± 0.02^ab^

*RC, relative crystallinity; To, Tp, Tc, and ΔH are onset temperature, peak temperature, conclusion temperature, and gelatinization enthalpy change, respectively*.

*Mean values followed by the same letter in the same row are not significantly different at the P < 0.05 level*.

Meanwhile, the results of thermal characteristics analysis showed that the onset temperature, peak temperature, and conclusion temperature decreased with the extension of germinating time, which indicated that wheat starch needed less energy to start gelatinization and had lower thermal stability to resist glass transition after germination. This was due to the destruction of amorphous areas during germination, which shortened the diffusion time of water in starch particles and accelerated starch gelatinization (33). The gelatinization enthalpy change (ΔH) (Table 1) reflected the melting of incomplete amylopectin-based crystals and may come from crystal accumulation and spiral melting enthalpy (34). The trend of ΔH showed that the longer the germination time, the less heat energy was required for the gelatinization process, which might be because the damaged amylopectin and starch granule fragments partly contributed to the endothermic gelatinization energy. UV-B radiation could inhibit the disintegration of the crystals formed by amylopectin, thus the ΔH slightly increased.

### Molecular Weight and Solubility of Proteins

**Figure 9:**
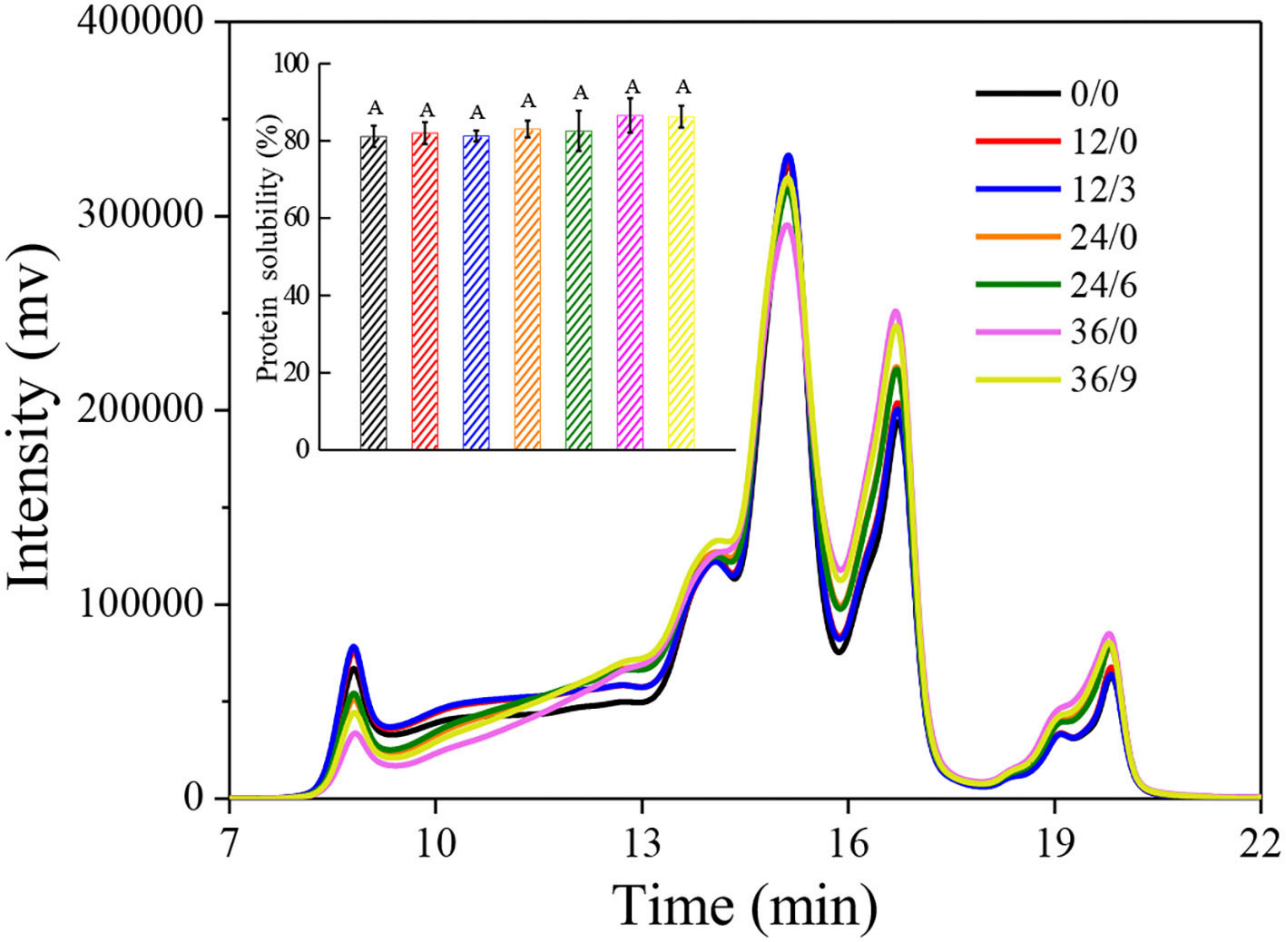
Size exclusion-HPLC chromatograms of whole wheat flour during germination under UV-B radiation. The inset shows protein solubility of wheat. Letter in the inset indicates the results of Duncan's multiple range test at 5% level of significant difference among different germination and UV-B radiation time, respectively.

Size exclusion high-performance liquid chromatography is a simple and efficient method for protein molecular weight fractionation. As shown in Figure 9, the large glutenin polymers in early germination (12 h) increased (8–10 min) which might be because the glutenin macropolymer was hydrolyzed to the large glutenin polymers at the beginning of germination. However, after more than 12 h of germination, wheat glutenin polymers were continuously degraded into monomeric proteins, short peptides, and free amino acids (16–20 min) by hydrolysis, which might be the reason for the decrease in G′ and G" in wheat after germination. But there was no significant increase in protein solubility (inset of Figure 9). Under UV-B radiation, protein hydrolysis was inhibited, which might be because UV-B radiation inhibited the germination of wheat, thus inhibiting protease activity and alleviating protein hydrolysis. The quantity and quality of wheat gluten determined the characteristics of wheat flour and thus determined the final wheat product quality. Therefore, UV-B radiation was advantageous to improve the processing quality of GW.

## Conclusion

Germination and UV-B radiation exerted a clear influence on the physicochemical properties, the accumulation of phenolic substances, and the dough-making quality of wheat. Under UV-B radiation, germination and dry matter loss of wheat were inhibited. Meanwhile, UV-B radiation improved the contents of free and bound phenolics (especially ferulic acid, *p*-coumaric acid, and sinapic acid), TPCs, AsA, and enhanced the DPPH radical-scavenging activity of GW. In addition, UV-B radiation inhibited the α-amylase activity, protein degradation process, and dough weakening and improved the mixing stability, so the dough-making quality of whole wheat flour during germination was enhanced. Therefore, as a benign inducer in plants, UV-B radiation could be an alternative approach to produce whole wheat flour with higher nutritional value and better dough-making quality.

## Data Availability Statement

The original contributions presented in the study are included in the article/supplementary material, further inquiries can be directed to the corresponding authors.

## Author Contributions

ML, MM, and CT provided research ideas and study design. YW, TY, and QS helped acquisition of data. MM and CT took care of data analysis and interpretation. ML and MM established critical revision of the manuscript for important intellectual content. All authors contributed to the article and approved the submitted version.

## Funding

This study was supported by the National Natural Science Foundation of China (32072135 and 32001614), Young Elite Scientists Sponsorship Program by CAST (2019QNRC001), the Program for Youth Science Innovation in Colleges and Universities of Shandong Province (2020KJF005), Advanced Talents Foundation of QAU (1120038), China Scholarship Council (201806850075), and the Open Project Program of China-Canada Joint Lab of Food Nutrition and Health, Beijing Technology and Business University (KFKT-ZJ-2106).

## Conflict of Interest

The authors declare that the research was conducted in the absence of any commercial or financial relationships that could be construed as a potential conflict of interest.

## Publisher's Note

All claims expressed in this article are solely those of the authors and do not necessarily represent those of their affiliated organizations, or those of the publisher, the editors and the reviewers. Any product that may be evaluated in this article, or claim that may be made by its manufacturer, is not guaranteed or endorsed by the publisher.

## References

[1] Calinoiu LF Vodnar DC. Whole grains and phenolic acids: a review on bioactivity, functionality, health benefits and bioavailability. Nutrients. (2018) 10:1615. 10.3390/nu10111615 30388881 PMC6265897

[2] Gomez M Gutkoski LC Bravo-Nunez A. Understanding whole-wheat flour and its effect in breads: a review. Compr Rev Food Sci Food Saf. (2020) 19:3241–65. 10.1111/1541-4337.12625 33337058

[3] Zhang Y Pei F Fang Y Li P Zhao Y Shen F. Comparison of concentration and health risks of 9 fusarium mycotoxins in commercial whole wheat flour and refined wheat flour by multi-Iac-Hplc. Food Chem. (2019) 275:763–9. 10.1016/j.foodchem.2018.09.127 30724260

[4] Cooper DN Kable ME Marco ML De Leon A Rust B Baker JE. The effects of moderate whole grain consumption on fasting glucose and lipids, gastrointestinal symptoms, and microbiota. Nutrients. (2017) 9:173. 10.3390/nu9020173 28230784 PMC5331604

[5] Chapa J Farkas B Bailey RL Huang JY. Evaluation of environmental performance of dietary patterns in the United States considering food nutrition and satiety. Sci Total Environ. (2020) 722:137672. 10.1016/j.scitotenv.2020.137672 32192968

[6] Hung PV Maeda T Yamamoto S Morita N. Effects of germination on nutritional composition of waxy wheat. J Sci Food Agric. (2012) 92:667–72. 10.1002/jsfa.4628 21919005

[7] Joshi P Varma K. Effect of germination and dehulling on the nutritive value of soybean. Nutr Food Sci. (2016) 46:595–603. 10.1108/NFS-10-2015-0123

[8] Xu M Jin Z Simsek S Hall C Rao J Chen B. Effect of germination on the chemical composition, thermal, pasting, and moisture sorption properties of flours from chickpea, lentil, and yellow pea. Food Chem. (2019) 295:579–87. 10.1016/j.foodchem.2019.05.167 31174798

[9] Michalcová E Potocká E Chmelová D Ondrejovič M. Study of wheat protein degradation during germination. J Microbiol Biotechnol. (2012) 1:1437–49.

[10] Takshak S Agrawal SB. Defense potential of secondary metabolites in medicinal plants under Uv-B stress. J Photochem Photobiol B. (2019) 193:51–88. 10.1016/j.jphotobiol.2019.02.002 30818154

[11] Ma M Wang P Yang R Gu Z. Effects of Uv-B radiation on the isoflavone accumulation and physiological-biochemical changes of soybean during germination: physiological-biochemical change of germinated soybean induced by Uv-B. Food Chem. (2018) 250:259–67. 10.1016/j.foodchem.2018.01.051 29412920

[12] Ma M Wang P Yang R Zhou T Gu Z. Uv-B Mediates isoflavone accumulation and oxidative-antioxidant system responses in germinating soybean. Food Chem. (2019) 275:628–36. 10.1016/j.foodchem.2018.09.158 30724242

[13] Chen Z Wang P Weng Y Ma Y Gu Z Yang R. Comparison of phenolic profiles, antioxidant capacity and relevant enzyme activity of different Chinese wheat varieties during germination. Food Bioscience. (2017) 20:159–67. 10.1016/j.fbio.2017.10.004

[14] Ma M Zhang H Xie Y Yang M Tang J Wang P. Response of nutritional and functional composition, anti-nutritional factors and antioxidant activity in germinated soybean under Uv-B radiation. Lwt. (2020) 118:108709. 10.1016/j.lwt.2019.108709

[15] Caceres PJ Martinez-Villaluenga C Amigo L Frias J. Maximising the phytochemical content and antioxidant activity of ecuadorian brown rice sprouts through optimal germination conditions. Food Chem. (2014) 152:407–14. 10.1016/j.foodchem.2013.11.156 24444955

[16] Pajak P Socha R Galkowska D Roznowski J Fortuna T. Phenolic profile and antioxidant activity in selected seeds and sprouts. Food Chem. (2014) 143:300–6. 10.1016/j.foodchem.2013.07.064 24054243

[17] Li M Sun QJ Han CW Chen HH Tang WT. Comparative study of the quality characteristics of fresh noodles with regular salt and alkali and the underlying mechanisms. Food Chem. (2018) 246:335–42. 10.1016/j.foodchem.2017.11.020 29291858

[18] Singh A Sharma S Singh B. Effect of germination time and temperature on the functionality and protein solubility of sorghum flour. J Cereal Sci. (2017) 76:131–9. 10.1016/j.jcs.2017.06.003

[19] Tavares DS Fernandes TEK Rita YL Rocha DC Sant'Anna-Santos BF Gomes MP. Germinative metabolism and seedling growth of cowpea (*Vigna unguiculata*) under salt and osmotic stress South African. J Bot. (2021) 139:399–408. 10.1016/j.sajb.2021.03.019

[20] Chen Z Ma Y Weng Y Yang R Gu Z Wang P. Effects of Uv-B radiation on phenolic accumulation, antioxidant activity and physiological changes in wheat (*Triticum aestivum* L) seedlings. Food Biosci. (2019) 30:100409. 10.1016/j.fbio.2019.04.010 30902306

[21] Hilal M Parrado MF Rosa M Gallardo M Orce L Massa EM. Epidermal lignin deposition in quinoa cotyledons in response to Uv-B radiation. Photochem Photobiol. (2004) 79:205. 15068034 10.1562/0031-8655(2004)079<0205:eldiqc>2.0.co;2

[22] Humphreys JM Chapple C. Rewriting the lignin roadmap. Curr Opin Plant Biol. (2002) 5:224–9. 10.1016/S1369-5266(02)00257-1 11960740

[23] Kaushik G Satya S Naik SN. Effect of domestic processing techniques on the nutritional quality of the soybean. Med J Nutrition Metab. (2010) 3:39–46. 10.3233/s12349-009-0079-7

[24] Xu M Dong J Zhu M. Effects of germination conditions on ascorbic acid level and yield of soybean sprouts. J Sci Food Agric. (2005) 85:943–7. 10.1002/jsfa.2050

[25] Salawu SO Bester MJ Duodu KG. Phenolic composition and bioactive properties of cell wall preparations and whole grains of selected cereals and legumes. J Food Biochem. (2014) 38:62–72. 10.1111/jfbc.12026

[26] Yao XQ Chu JZ He XL Si C. The effects of Uv-B radiation intensity on biochemical parameters and active ingredients in flowers of Qi chrysanthemum and huai chrysanthemum. Photochem Photobiol. (2014) 90:1308–13. 10.1111/php.12329 25112378

[27] Wang P Liu K Yang R Gu Z Zhou Q Jiang D. Comparative study on the bread making quality of normoxia- and hypoxia-germinated wheat: evolution of gamma-aminobutyric acid, starch gelatinization, and gluten polymerization during steamed bread making. J Agric Food Chem. (2019) 67:3480–90. 10.1021/acs.jafc.9b00200 30817141

[28] Patraşcu L Banu I Vasilean I Aprodu I. Rheological and thermo-mechanical characterization of starch–protein mixtures. Agriculture and Agricultural Science Procedia. (2016) 10:280–8. 10.1016/j.aaspro.2016.09.065

[29] Bhat NA Wani IA Hamdani AM Masoodi FA. Effect of gamma-irradiation on the thermal, rheological and antioxidant properties of three wheat cultivars grown in temperate Indian climate. Radiat Phys Chem. (2020) 176:108953. 10.1016/j.radphyschem.2020.108953

[30] Wang P Liu K Gu Z Yang R. Enhanced gamma-aminobutyric acid accumulation, alleviated componential deterioration and technofunctionality loss of germinated wheat by hypoxia stress. Food Chem. (2018) 269:473–9. 10.1016/j.foodchem.2018.07.050 30100462

[31] Mares D Mrva K. Late-Maturity α-Amylase: low falling number in wheat in the absence of preharvest sprouting. J Cereal Sci. (2008) 47:6–17. 10.1016/j.jcs.2007.01.005

[32] Ding J Hou GG Nemzer BV Xiong S Dubat A Feng H. Effects of controlled germination on selected physicochemical and functional properties of whole-wheat flour and enhanced gamma-aminobutyric acid accumulation by ultrasonication. Food Chem. (2018) 243:214–21. 10.1016/j.foodchem.2017.09.128 29146331

[33] Wang S Copeland L. Phase Transitions of pea starch over a wide range of water content. J Agric Food Chem. (2012) 60:6439–46. 10.1021/jf3011992 22667995

[34] Chen X He X Fu X Huang Q. *In vitro* digestion and physicochemical properties of wheat starch/flour modified by heat-moisture treatment. J Cereal Sci. (2015) 63:109–15. 10.1016/j.jcs.2015.03.003

